# Differences in moral reasoning among medical graduates, graduates with other degrees, and nonprofessional adults

**DOI:** 10.1186/s12909-022-03624-z

**Published:** 2022-07-23

**Authors:** M. G. Jean-Tron, D. Ávila-Montiel, H. Márquez-González, G. Chapa-Koloffon, J. A. Orozco-Morales, A. V. Ávila-Hernández, O. Valdés-Pérez, J. Garduño-Espinosa

**Affiliations:** 1 Department of Clinical Research, Hospital Infantil de México Federico Gómez, Mexico City, Mexico; 2 Medical Sciences of the Master and Doctorate Program in Medical, Odontological and Health Sciences, National Autonomous University of Mexico, Mexico City, Mexico; 3 Hospital Infantil de México Federico Gómez, Tercer piso Edificio Hemato-Oncología e Investigación, Calle Dr. Márquez 162, Col. Doctores, CP 06720, Cuauhtémoc, Mexico City, Mexico; 4 Hospital Infantil de México Federico Gómez, Mexico City, Mexico

**Keywords:** Moral, Reasoning, Education, Professional

## Abstract

**Background:**

Reasoning and moral action are necessary to resolve day-to-day moral conflicts, and there are certain professions where a greater moral character is expected, e.g., medicine. Thus, it is desirable that medical students develop skills in this field. Some studies have evaluated the level of moral reasoning among medical students; however, there are no comparative studies involving other types of populations. Therefore, the objective of this study was to compare the moral reasoning among medical graduates with that of a group of young graduates with other degrees and of a group of nonprofessional adults.

**Methods:**

An exploratory cross-sectional study was conducted. Pediatric residents and pediatric subspecialty residents at a pediatric hospital were invited to participate, forming the group of “medical graduates”. A group of young people from a social program and students with a master’s degree in a science from the same pediatric hospital were also invited to participate, comprising the group of “graduates with other degrees”. Finally, a group of beneficiaries of a family clinic was invited to participate, which we categorized as “nonprofessionals”. To evaluate the differences in moral reasoning between these 3 groups, we applied the Defining Issues Test (DIT), a moral reasoning questionnaire designed by James Rest using Kohlberg’s theory of moral development.

**Results:**

The moral reasoning of 237 subjects—88 from the “medical graduates” group, 82 from the “graduates with other degrees” group and 67 from the “nonprofessionals” group— was evaluated. We found differences in the profiles of moral development of the groups. The profile of the “nonprofessionals” showed a very high predominance of subjects at the preconventional level, 70%, but only 4.5% at the postconventional level. Among the “medical graduates”, we observed 37.5% at the preconventional level and 34% at the postconventional level (X^2^ *p* < 0.001); this group had the highest percentage in this category. This large difference could be because the differences in the ages and socioeducational levels of nonprofessionals are much wider than those among medical graduates. However, significant differences were also found when the profiles of medical graduates were compared with those of graduates with other degrees, since the latter demonstrated 56% at the preconventional level and 18% at the postconventional level (X^2^ test, *p* = 0.02).

**Conclusions:**

Significant differences were found in moral reasoning among the groups that we evaluated. Among the group of medical graduates, there was a higher percentage of subjects at the postconventional level than among the group of graduates with other degrees and a much higher percentage than among the group of nonprofessionals. Our conclusions give the first evidence that studying medicine seems to influence the development of moral reasoning in its students. Therefore, we consider it relevant to develop educational strategies where the student is involved in simulated but realistic decision-making situations, where there are moral dilemmas to resolve from their early years of training.

## Introduction

Moral reasoning is defined by Kohlberg as judgment about what is right and wrong. Although there are multiple definitions for this concept, this characterization will be used for the purposes of this research. Kohlberg’s developmental cognitive development approach proposes that morality follows universal moral principles that are not learned in early childhood and are the product of mature rational judgment [1–3]. Notably, “the exercise of morality is not limited to rare moments in life; it is an integral part of the thought process that we use to extract meaning from the moral conflicts that arise in daily life” [2, 4]. According to Kohlberg’s theory, there are 6 stages of progressive moral reasoning, encompassed in 3 levels. Individuals with a low level of moral reasoning (preconventional level, stages 1 and 2) judge moral issues using a scheme of personal interest. Those with a medium level (conventional level, stages 3 and 4) judge them using compliance with rules and norms, while individuals at the highest level (postconventional level, stages 5 and 6) judge moral issues using universal principles and shared ideals [1, 3, 5].

The most commonly used instrument to evaluate moral reasoning has been the *Defining Issues Test* (DIT), which was developed using Kohlberg’s theory of moral development [6]. This instrument has been used in multiple descriptive studies regarding a similar profile of moral reasoning among their subjects, where an increase in the score from stage 2 to 4 has been found to subsequently decrease in the higher stages, although some differences have been observed in the averages of the moral reasoning of principles (P index) among individuals [7–9]. The main variables used to explain such differences have been age, educational level and socioeconomic status but not sex [6, 10].

There are certain professions, e.g., medicine, where greater professionalism and certain qualities are expected. Of these, a high moral character stands out due to the day-to-day decision-making required by professional medical practice concerning the life and health of other people [11]. Thus, as researchers, we are interested in whether a medical career influences a young person to develop a high level of moral reasoning. Several studies have evaluated moral reasoning among both students and medical professionals [8, 9, 12–16], finding differences between internal physicians’ PI (PI = 37.2) and the PI of surgical residents (PI = 46) [12]. These could suggest that with more years of experience in medical practice, a greater level of moral reasoning results, entailing less aggressive treatment of neonates with malformations [13], which suggests that developing a high level of moral reasoning improves the quality of care, i.e., the beneficial treatment of patients. Similar studies have been carried out among other professionals; a study of veterinarians found no differences between their moral reasoning and that of the general public [17], suggesting that having a professional degree does not improve moral reasoning. Moreover, a study of pharmaceutical students found a lower PI than the authors expected (PI = 25.21) [18]. Accordingly, our objective in the present study was to compare the levels of moral reasoning of medical graduates, young graduates with other degrees and nonprofessional adults. Secondarily, we also evaluated differences in moral reasoning by sex, age and educational level.

## Methodology

A cross-sectional exploratory comparative study was conducted from August 2019 to February 2020. To form the group of “medical graduates”, pediatric residents and pediatric subspecialty residents enrolled at a third-rate children’s hospital were invited to participate. Of the 345 enrolled in 2019–2020, 99 subjects (29%) agreed to participate. To form the group of “graduates with other degrees”, young people enrolled in a social program of the Mexican government who were completing internships at a tertiary children’s hospital and who met the criterion of having graduated were invited to participate. In addition, those enrolled in the prerequisite curriculum of the Master of Health Sciences program based at the same hospital were invited to participate. Based on this selection criteria, 107 (88%) agreed to participate. Finally, to form the group of” “nonprofessionals”, the beneficiaries of a family clinic in Mexico City who visited for consultation during the study period and met the criterion of being over 18 years of age were invited to participate. Those with a bachelor’s degree or higher were excluded; 98 subjects agreed to participate. After participants agreed to participate and signed the informed consent letter, they received 2 questionnaires. The first concerned general data including age, sex, place of residence, religion, monthly income and other sociodemographic data.

The second questionnaire was the DIT moral reasoning questionnaire, designed by James Rest in 1979 using Kohlberg’s theory [19]. It has been validated in Mexico and other Latin American countries. The results show adequate internal consistency, with a Cronbach’s alpha of 0.71, and test–retest procedures of 0.65 [7, 20]. These results are similar to those obtained by Rest, who used the DIT in its original version; his Cronbach’s alpha coefficient was approximately 0.70, with a test–retest reliability of between 0.70 and 0.80 [19]. The DIT has 6 stories in its original version and 3 stories in its short version, which was used for this study. Each of the 3 stories presented the subjects with a moral dilemma. In the first section, the subjects were asked their opinion about what the person in the story should do, and they were able to answer “yes”, “no” or “I cannot decide”. In the second section, the subjects were asked to give their opinion regarding the degree of importance of 12 statements, which represented each of the moral stages of Kohlberg. The statements offered possible resolutions to the dilemmas. In the third section, the subjects selected the 4 most important statements of each story, using what they had chosen in the second section, and then ranked them from first to fourth place in decreasing order of importance. By evaluating these responses and the statements that the subjects gave greater importance to, we generated raw and percentage scores (scale from 0 to 100) to express the frequency with which the subjects used each of stages 2 to 6 of moral reasoning.

The M score, i.e., the score obtained from assigning much importance to “nonsense” statements, was also calculated. A high M score entails that a subject does not qualify for any stages of moral reasoning; if an M score is greater than 16, the questionnaire must be invalidated. Additionally, PI was calculated, expressing the degree to which a person judges moral problems from a postconventional perspective. The PI was prepared with scores corresponding to stages 5a, 5b and 6 and was expressed on a scale from 0 to 100. In our analyses, medians were therefore calculated for each stage and for the PI.

Moral development profiles were developed using medians and minimum–maximum ranges because not all groups met a normal distribution. We calculated the medians for stages 2 to 6 and the PI of the total population and of each group. PI has been the most used indicator to evaluate the moral reasoning of subjects since it reflects the tendency to use postconventional reasoning. The higher the PI score is, the greater the tendency to use this type of reasoning; the lower the score is, the greater the tendency to use preconventional or conventional reasoning. Thus, this index can be used to make categories of the level of moral reasoning with the following thresholds: the preconventional level corresponds to a PI of less than 30 points, the conventional level to a PI between 30 and 40 points and the postconventional level to a PI greater than 40 points [19]. In this study, we calculated the frequencies of each of the levels of moral reasoning.

A descriptive analysis was performed on the sociodemographic characteristics of our study population. The qualitative variables were reported as total numbers and percentages, and the quantitative variables were reported as the mean and standard deviation. To evaluate the differences between the groups of both the medians of stages 2 to 6, as well as the PI, we used the Kruskal–Wallis test. We also analyzed the differences among “medical graduates” and “graduates with other degrees” using the Mann–Whitney U test. To evaluate the differences between all the groups in terms of their levels of moral reasoning, the X [2] test was used, and the results were considered statistically significant at *p* < 0.5. To evaluate whether age was correlated with PI, a Pearson correlation was performed for all subjects and by group. Microsoft Excel was used to generate our database with the sociodemographic variables, the quantitative results of the DIT (scores from stages 2 to 6, M score and PI) and the qualitative results of the DIT (level of moral reasoning). SPSS v24 software was used for statistical analysis.

## Results

In total, 304 questionnaires were completed, and 67 (22%) of these were invalidated, comprising a total of 237 questionnaires: 88 from the group of medical graduates, 82 from the group of graduates with other degrees and 67 from the nonprofessional group. A total of 45 questionnaires were invalidated due to incomplete completion, 17 due to inconsistencies and 5 due to exceeding the allowed M score.

Table 1 describes the total and group sociodemographic characteristics of the 237 final participants, where a predominance of females can be observed in all 3 groups. The mean age was 32.5 years, with a significantly higher mean age in the nonprofessional group (44.2) than in the other 2 groups (26.4 and 29.4). The majority of participants (65%) were single, and 28% were married. A total of 66% reported having some type of religion; however, only 53% of these professed this. Regarding income level, 35% of the subjects reported having income between $699 and $2700 and 30% between $6800 and $11,599. There were thus significant income differences, as we expected, between the groups, with a higher income level among medical graduates than nonprofessionals.

**Table 1:** Sociodemographic characteristics of the total population and each group

	Total *n* = 237 NT (%)	Medical graduates *n* = 88 NT (%)	Graduates with other degrees *n* = 82 NT (%)	Nonprofessionals *n* = 67 NT (%)
Female	165 (69.6)	61 (69.3)	58 (70.7)	46 (68.7)
Male	72 (30.4)	27 (30.7)	24 (29.3)	21 (31.3)
**Age**^**a**^ (mean ± SD)	32.5 ± 13.3	26.4 ± 2.4	29.4 ± 9.8	44.2 ± 17.4
Marital Status*:				
Single	156 (65.3)	82 (93.2)	59 (72)	15 (22.4)
Married or Free union	66 (27.6)	5 (5.7)	20 (24.4)	41 (61.2)
Divorced or Separated	10 (4.2)	1 (1.1)	3 (3.7)	6 (9)
Widower	5 (2.1)	0	0	5 (7.5)
**Religion**	160 (67.5)	54 (61.4)	54 (65.8)	52 (77.6)
Practice religion* (*n* = 160)	85 (53.1)	25 (46.3)	20 (37)	40 (76.9)
Academic level*				
Primary	9 (3.8)	0	0	9 (13.4)
Secondary	22 (9.2)	0	0	22 (32.8)
High School	36 (15.1)	0	0	36 (53.7)
Bachelor	147 (62)	83 (94.3)	64 (78)	0
Postgraduate	23 (9.7)	5 (5.7)	18 (22)	0
Monthly income*:				
0–2699	32 (13.4)	8 (9.1)	14 (17.1)	10 (14.9)
2700-6799	84 (35.1)	10 (11.4)	26 (31.7)	48 (71.6)
6800-11,599	71 (29.7)	46 (52.3)	17 (20.7)	8 (11.9)
11,600-34,999	39 (16.3)	18 (20.5)	20 (24.4)	1 (1.5)
Más de 35,000	11 (4.6)	6 (6.8)	5 (6.1)	0

*NT* Total numbers, *CDMX* Mexico City.

^a^ ANOVA *p* < 0.001

*X^2^ *p* < 0.05

### Level of moral reasoning analysis

Table 2 shows the results of the development of moral reasoning using the DIT of both the participants and each group. We observed that the scores increase in the first stages until reaching a higher score in stage 4, with a median of 36.9, and a decrease in the scores as the stages increase. This trend is repeated in each of the groups; however, there were significant differences between the groups. The group of medical graduates had a larger score in the high stages (5ª and 5b) than the other 2 groups. The PI showed a median of 29.2, with significant differences between the groups, i.e., a higher score (33.3) for medical graduates than the group of graduates with other degrees (PI = 26.6) and nonprofessionals (PI = 20) (Kruskal–Wallis *p* < 0.001).

**Table 2:** Medians and minimum–maximum ranges of stages 2 to 6 and PI for the total population and each group and frequencies of total and group moral reasoning levels

Stages	Total *n* = 237 med (min–max)	Graduates from medicine *n* = 88 med (min–max)	Graduates with other degrees *n* = 82 med (min–max)	Nonprofessionals *n* = 67 med (min–max)	p (Kruskal–Wallis)
2	6.1 (0–26.6)	5 (0–23.3)	3.3 (0–26.6)	3.3 (0–26.6)	*p* = 0.68
3	17.3 (0–53.3)	13.3 (0–53.3)	20 (0–50)	16.6 (0–43.3)	***p*** **= 0.04**
4	36.9 (0–80)	36.6 (0–80)	35 (6.6–63.6)	36.6 (0–70)	*p* = 0.14
5A	17.4 (0–56.6)	20 (0–56.6)	16.6 (0–56.6)	13.3 (0–36.6	***p*** **< 0.001**
5B	5.6 (0–13.6)	10 (0–13.3)	6.6 (0–13.6)	0 (0–13.3)	***p*** **< 0.001**
6	6.5 (0–20)	6.7 (0–20)	6.6 (0–20)	6.6 (0–20)	*p* = 0.27
PI	29.2 (3.3–83.3)	33.3 (0–76.6)	26.6 (3.3–83.3)	20 (0–50)	***p*** **< 0.001**
N. preconventional*	126 (52.7)	33 (37.5)	46 (56.1)	47 (70.1)	***p*** **< 0.001**^**a**^
N. conventional*	61 (25.5)	23 (26.1)	21 (25.6)	17 (25.4)	
N. postconventional*	50 (20.9)	32 (36.4)	15 (18.3)	3 (4.5)	

*NT (%): Total numbers

^a^X^2^

Regarding the levels of moral reasoning, 53% of the population was at the preconventional level, 25% at the conventional level and only 21% at the postconventional level. When evaluating the differences between the groups, we noted a higher proportion of subjects at the postconventional level in the group of medical graduates (36.4%) than among graduates with other degrees (18.3%) and nonprofessionals (4.5%; X ^2^ *p* < 0.001). In contrast, a greater proportion of subjects with a preconventional level was found among nonprofessionals (70.1%) than graduates with other degrees (56%) and medical graduates (37.5%) (Table 2).

We also found specific differences between the group of medical graduates and the group of graduates with other degrees. Through the Mann–Whitney U test, significant differences were calculated for the PI (*p* < 0.01) and scores of stages 3 and 5a (*p* < 0.05). Regarding the categories of levels of moral reasoning, a statistically significant difference was also found with the X ^2^ test (*p* = 0.02).

### Results of the level of moral reasoning analysis with other variables

When analyzing the differences in moral reasoning between the sexes, a difference of almost 7 points was found for the PI medians between men (PI = 23.3) and women (PI = 30) (Mann–Whitney U *p* = 0.017). When evaluating the levels of moral reasoning, we observed that 64% of men were within the preconventional level and only 17% were within the postconventional level. In contrast, 49% of women were in the preconventional level and 23% in the postconventional level, although these percentages were not statistically significant (X ^2^ *p* = 0.09).

When accounting for the level of education of the participants, the median P-score increased as the highest grade increases. Primary school PI = 16.6, secondary school PI = 21.7, high school PI = 20 until obtaining bachelor’s degree, where PI = 30. However, a slight decrease was observed among the participants whose PI = 26.7 (Kruskal–Wallis *p* = 0.001). The same occurs with level of moral reasoning, reaching 89% in the preconventional level among the participants with only elementary schooling, 72% among those with high school education and only 47% in those with bachelor’s degrees. In contrast, at the postconventional level, no participants with primary or secondary school education were observed; those with a high school education comprised 12%, increasing to 27% among those with bachelor’s degrees (27%) and decreasing among those with postgraduate degrees (17%) (X ^2^ *p* = 0.016).

Concerning age, we performed a correlation analysis between age and the PI of the subjects, finding a low negative correlation (*r* = − 0.21 *p* = 0.001). That is, the higher the age is, the lower the PI score. The ages varied between the groups, and thus we decided to perform an analysis for each of the groups. However, we found no significant correlations in any of them.

Regarding whether the participants reported having a religion or not, the PIs did not reflect any differences: 26.7 points for those belonging to some religion and 26.6 for those who did not. Only those who did belong to a religion (157 subjects) were asked whether they frequently practiced their religion or not. The PI of those who did not regularly practice was 30 points, while that of those who did practice was 26.6 (Mann–Whitney U *p* = 0.019). Among those who did, 58% were found at the preconventional level, 27% at the conventional level and 15% at the postconventional level. Of those who did not, 42% were observed at the preconventional level, 35% at the conventional level and 23% at the postconventional level (X ^2^ *p* = 0.15).

## Discussion

The present study reveals the differences in the levels of moral reasoning among medical graduates, graduates with other degrees, and nonprofessionals.

The moral development profile of the total population studied showed a predominance of stage 4, which corresponds to a conventional level of moral reasoning, that is, a norm-keeping scheme of Kolhberg’s theory of moral development, this same profile has been reported by other authors [8, 17, 21].. The average PI score of the population was 29.2, similar to other studies of the Mexican population. Barba, for example, found an average P index of 25.9 for a group of upper-level students and showed that students older than 21 years had a PI of 28.2, which was higher than the approximately 24.1 points of younger students. Similarly, secondary and high school students generated a lower PI of approximately 21.1; hence, age and academic level could influence the type of moral reasoning.^7.22^ Regarding the level of moral reasoning, our total studied population showed a clear predominance of preconventional reasoning, almost 53%, with only 21% of the participants falling into the postconventional level.

The differences found in the levels of moral reasoning between the groups, specifically in the postconventional level with a higher percentage of subjects in the group of medical students and the differences in the P index could be due to the fact that the differences in age, social and educational level of non-professionals are wider than those of medical graduates. However, when the profiles of the latter are compared to those who graduated with other degrees, significant differences were found; the latter had 56% of subjects at the preconventional level and 19% at the postconventional level. Moreover, there was a statistically significant difference in these groups’ PIs (graduates with other degrees’ PI = 26.6, medical graduates’ PI = 33.3).

To evaluate whether there were other variables that could influence our results, we analyzed the sex, age, academic level and religion. We found differences in these results with the sex variable, since women showed a higher PI score (PI = 30) than men (PI = 23.3). This could contradict Kolhberg [3], who suggested that men tend to use greater moral reasoning involving principles (postconventional reasoning) than women. Nevertheless, several empirical studies have shown the opposite, e.g., Barba’s 2003 and 2005 studies [7, 22], whose results correspond to our findings. Other authors, however, have not found differences between the sexes [8, 12, 17, 21].

Regarding age, we found that at an older age, there was a lower PI and therefore a lower tendency to use postconventional moral reasoning (*r* = − 0.21 *p* = 0.001). Notably, our evaluated groups showed significant differences for this variable, given that the average age of the medical graduates was 26.4 years and that of the nonprofessional group was 44.2 years. In the latter group, moreover, the subjects with the lowest PI level were found. Education, a variable with greater weight to determine the level of moral reasoning [3, 17, 22], seems to entail that age has a lower influence. To corroborate this information, we performed a correlation analysis between age and PI for only the group of nonprofessionals and found no correlation.

According to the maximum levels of other studies, we also found differences in the profiles of moral development. Regarding the percentage of subjects at the postconventional level, this seems to increase according to a higher degree of education, with 0% in primary and secondary, 12% in secondary, 27% in undergraduate. Strikingly, among subjects with postgraduate degrees, this percentage decreases to 17%. This increase in the level of moral reasoning while advancing in educational level has already been described by other authors [7, 17, 22], corroborating our findings. On the other hand, the anomaly found among the undergraduate and graduate subjects could be explained by their degree topics, since the majority of the subjects with postgraduate degrees in the total sample (18/23) belonged to the group of graduates with other degrees; only 5 of the 23 were in the group of medical graduates. We also evaluated the religion variable and found no significant differences in the profiles of moral development.

Our findings seem to confirm the hypothesis that a chosen career influences the level of moral reasoning of the subject; specifically, studying medicine seems to influence the development of this type of reasoning, possibly because these students consistently face ethical dilemmas in their clinical practice. Although some authors have found failures of medical education to develop moral reasoning in students [9, 16], there is actually little evidence for this. Indeed, our findings show a clear difference between medical graduates and those with other degrees.

In a certain way, doctors are expected to have greater professionalism and ethics than other professionals. Medical professionalism is understood as placing the patient’s interest above that of the doctor, always maintaining standards of competence and integrity, as well as providing expert advice to society in health matters, to achieve this requires that the doctor respect the ethical principles of patient well-being and autonomy as well as social justice [23], for a person to understand and follow these principles it is necessary to develop moral reasoning of a post-conventional type, that is why we consider it important that the future professional of medicine develops it and that medical schools give moral reasoning the importance it has as a fundamental part for professionalism in medical practice and therefore encourage it in their students.

From a sociological point of view, the health professional is not only the agent who has formal knowledge, but also the one who has the ability to serve the needs of the other, as well as for the common good and society [24], from an economic perspective medicine is considered an imperfect market due to the asymmetry of information between doctor and patient, which means that strict control and adherence of the professional to an ethical code of conduct are required in medicine [25].

This is transmitted since the doctor is a student and has to deal with dilemmas that put the doctor’s actions to the test where these ethical principles must be taken into account for the correct decision making, at the beginning observing their teachers and then do the exercise themselves under supervision. This continuous observation and making of “decision making” may be the reason why we have observed a greater development of moral reasoning in medicine graduates than in non-professionals and even in graduates of other degrees. Since it is finally known that to make the best decisions, it is not only necessary to have the knowledge, but it is necessary adequate reasoning and in cases where a moral dilemma is involved, moral reasoning is indispensable and it is with the practice of the day to day that this type of more post-conventional reasoning can be consolidated, which is what could be expected of medical professionals, leaving behind the making of moral decisions of a more conventional or conventional nature, where, in the latter, it would be seen first by one’s own interests before that of others, and in the former one would only be following rules, sometimes without internalizing the importance of them.

Studies have found that the hidden curriculum is involved in the formation of professional ethics, that is, students learn from what they see [26], so that despite the fact that the development of ethics in medical students is one of the responsibilities of medical education and in some universities it is done through formal ethics courses [27], we consider it important that medical schools continue to encourage reasoned decision-making in their students, always considering fundamental ethical principles, in simulated but realistic situations where there are moral dilemmas to resolve and do so through strategies that include role-playing and cooperative learning, with someone facilitating an appropriate discussion [28].

Another possible explanation is that people who choose careers such as medicine differ in terms of their moral development, from the beginning, from people who decide to dedicate themselves to other trades or professions.

It is known that there are both innate and acquired factors that influence the socio-emotional development of people and that the first years of life are of paramount importance [29–31] and probably also influence moral development.

However, many of the students who enter a university career are still developing when entering at the ages of 18 or 19, so the environmental influence, specifically the educational one, that they have in those years, can be decisive for their moral development.

The study of moral reasoning at younger ages (late adolescents) will undoubtedly be useful in defining whether there are associations between it and the selection of different types of university careers and this information could be relevant to select students in medical schools.

Concerning the limitations of the study, there were very important differences in age among the groups. Our findings with this variable are thus biased, and it cannot be concluded that age negatively influences the PI of moral reasoning. On the other hand, since this is a cross-sectional study, it cannot be determined whether the level of moral reasoning of the subjects has been stable over time or was not altered for any reason at the time of measurement (via fatigue, worry, etc.). Moreover, since we used convenience sampling, people who were performing practices and studies at the same location were recruited (in the case of medical graduates and graduates with other degrees) as were people who attended a specific family clinic a part of Mexico City with particular demographic characteristics. Accordingly, it is not possible to ensure our findings’ representativeness of other types of populations.

## Conclusion

The results of this study indicate significant differences in moral reasoning among the groups we evaluated. Among the group of medical graduates, there was a higher percentage of subjects at the postconventional level with a higher PI than the group of graduates with other degrees and a much higher percentage than the nonprofessional group. Although the educational level, per se, can influence the development of moral reasoning, our findings give evidence, that studying medicine may influence the development of moral reasoning in its students, who model in terms the ethical decision-making of their teachers in regular clinical practice plays an important role and therefore we consider important to continue promoting this type of education by developing strategies where the student is involved in simulated but realistic situations of medical decision-making, where there are moral dilemmas to resolve.

## Data Availability

The datasets generated and/or analysed during the current study are not publicly available due to the sensitive nature of the data but are available from the corresponding author on reasonable request.

## Abbreviations

DIT: Defining issues test
UMF: Family medicine clinic
JCF: Young people enrolled in a social program of the mexican government.
Av: Average
TN: Total numbers
